# One-dimensional mid-IR defect-mode photonic crystal biosensor for oral cancer detection

**DOI:** 10.1038/s41598-026-59748-5

**Published:** 2026-06-30

**Authors:** Arafa H. Aly

**Affiliations:** TH-PPM group, Physics department, Faculty of Sciences, Beni-Suef University, Beni Suef, 62111 Egypt

**Keywords:** Graphene-assisted photonic crystal, Oral cancer sensor, Mid-infrared biosensor, Defect-mode resonance, Transfer matrix method, Refractive-index sensing, Optical biosensor, Photonic band gap, Field localization, One-dimensional photonic crystal, Optics and photonics, Physics

## Abstract

A graphene-assisted one-dimensional photonic crystal biosensor for oral cancer detection in the mid-infrared region is theoretically proposed and analyzed using the transfer matrix method. The designed structure consists of alternating high- and low-refractive-index dielectric layers surrounding an oral-tissue defect cavity that supports a highly localized defect-mode resonance inside the photonic band gap. Variations in the refractive index of healthy and cancerous oral tissues modify the optical path length of the cavity and induce measurable resonance wavelength shifts, forming the basis of the sensing mechanism. The proposed biosensor exhibits a high refractive-index sensitivity of 1629.82 nm/RIU with excellent linearity (R^2^ = 0.9997). In addition, the structure demonstrates a narrow resonance linewidth with an average full width at half maximum of 17.01 nm and a high quality factor of 470.25. The obtained figure of merit, detection accuracy, and limit of detection are 95.81 RIU^− 1^, 0.0588 nm^− 1^, and 0.0104 RIU, respectively. Reflectance and transmittance analyses confirm the formation of a stable localized defect mode, while electric-field distribution maps reveal strong electromagnetic confinement inside the oral-tissue cavity, leading to enhanced light–matter interaction and improved sensing performance. Furthermore, angular-response analysis, defect-thickness optimization, and fabrication-tolerance evaluation demonstrate the robustness and stability of the proposed design under practical operating conditions. The obtained results indicate that the proposed graphene-assisted photonic crystal biosensor provides a promising platform for highly sensitive and reliable oral cancer detection in the mid-infrared spectral region.

## Introduction

Oral cancer is one of the most prevalent malignancies affecting the head and neck region and continues to represent a serious clinical challenge because of its high mortality rate and frequent late-stage diagnosis^1–3^. Despite considerable progress in therapeutic strategies, early detection remains the most critical factor for improving patient survival and reducing disease progression. Conventional diagnostic techniques such as visual inspection, imaging, histopathological examination, and biopsy are clinically important; however, they may suffer from invasiveness, sampling limitations, subjective interpretation, and insufficient sensitivity for detecting subtle early-stage abnormalities^4–6^. Consequently, there is an increasing demand for compact, rapid, and label-free optical sensing platforms capable of detecting small biological variations associated with cancerous transformation. Cancer development is commonly accompanied by structural, biochemical, and morphological alterations in biological tissues, leading to measurable changes in their optical properties, particularly the refractive index^7–9^. Refractive-index-based optical biosensors have therefore attracted significant attention because they can translate minute changes in the analyte medium into detectable optical responses^10–12^. Among different optical sensing technologies, photonic crystal (PhC)-based biosensors have emerged as highly promising platforms due to their capability to manipulate electromagnetic-wave propagation through photonic band-gap engineering and strong light confinement^13–16^. In defect-mode photonic crystals, introducing a defect cavity into a periodic multilayer structure generates a localized resonant mode inside the photonic band gap. The resonance wavelength strongly depends on the optical properties of the defect layer, making such structures highly suitable for biological and chemical sensing applications. The mid-infrared (mid-IR) spectral region is particularly attractive for biomedical sensing because many biological molecules exhibit characteristic optical responses in this range^17^. Recently, different optical biosensing strategies have been explored for biomedical and biochemical detection, including surface plasmon resonance sensors, multilayer optical biosensors, graphene-assisted sensing structures, and machine-learning-assisted photonic devices^18–30^. Surface plasmon resonance configurations have demonstrated high sensitivity for pregnancy detection, glucose monitoring, and anemia diagnosis^18,19^. In addition, compact photonic-crystal biosensors have been proposed for biomedical sensing applications, including two-dimensional cavity biosensors and oral-cancer detection systems^20,29,30^. One-dimensional photonic crystal biosensors have also shown promising performance for biochemical sensing because of their compact geometry, narrow resonance linewidth, and tunable optical response^21^. Theoretical analysis of multilayer optical systems using transfer matrix formalism has further provided an efficient framework for analyzing resonance behavior and wave propagation in layered photonic structures^22,23^. Moreover, graphene- and transition-metal-based materials such as MoS₂ and WS₂ have been integrated into optical biosensors to improve field confinement and sensitivity enhancement^24,25,28^. Recent studies have also explored phase-change materials, GaN-based photonic crystal structures, and machine-learning-assisted biosensing approaches for disease detection and biomedical monitoring^26,27^. Several advanced photonic crystal sensing architectures have been reported in the literature, including nanophotonic resonator-assisted sensing platforms, optical Tamm-state structures, photonic crystal microcavities, chirped graded resonators, slotted waveguides, optimized H1 cavities, and one-dimensional defect-mode photonic crystals^31–43^. Goyal and co-workers investigated photonic spin Hall enhancement using nanophotonic resonators, dynamically tunable optical Tamm states, photonic crystal microcavity sensors, chirped resonators, DAST-assisted tunable filters, and waveguide-based refractive-index sensing structures^31–36^. Kassa-Baghdouche and co-workers further demonstrated optimized H1 microcavities, slotted photonic crystal waveguides, and defect-mode photonic crystal structures for gas and refractive-index sensing in visible and mid-infrared spectral regions^37–43^. These studies confirm the strong capability of photonic crystal structures for enhancing resonance confinement, electromagnetic localization, and refractive-index sensitivity.

However, many previously reported photonic crystal sensors rely on relatively complex two-dimensional geometries, slotted waveguides, or advanced resonator configurations that may require demanding fabrication procedures and sophisticated numerical optimization. In contrast, one-dimensional defect-mode photonic crystals provide a simpler multilayer geometry that can be efficiently analyzed using the transfer matrix method while allowing flexible control of resonance wavelength, linewidth, Q-factor, and structural tolerance through layer thickness and refractive-index engineering. Such structures are also more compatible with thin-film fabrication techniques and practical optical integration. In the present work, a graphene-assisted one-dimensional defect-mode photonic crystal biosensor operating in the mid-infrared region is theoretically investigated for oral-cancer-related refractive-index detection. The oral tissue is modeled as an effective defect cavity whose refractive index changes between healthy and cancerous conditions. The sensing mechanism is based on monitoring the resonance wavelength shift of the localized defect mode inside the photonic band gap. Unlike many previous studies that mainly focus on sensitivity enhancement alone, the proposed structure is evaluated through a comprehensive multi-parameter analysis including resonance shift, linearity, full width at half maximum, Q-factor, figure of merit, detection accuracy, limit of detection, dip depth, field localization, angular stability, defect-thickness optimization, and fabrication-tolerance analysis. The obtained results demonstrate strong electromagnetic confinement, highly linear spectral response, and balanced sensing performance, confirming the potential of the proposed structure as a compact and efficient optical platform for oral-cancer-related biosensing.

## Theoretical background and performance metrics

The proposed biosensor is based on a one-dimensional defect-mode photonic crystal designed to operate in the mid-infrared spectral region. The structure consists of a graphene-assisted entrance layer followed by alternating high- and low-refractive-index dielectric layers arranged around a central defect cavity filled with the oral-tissue analyte. The general configuration can be written as:

### Air / G / (HL)^N^ / D / (LH)^N^ / Substrate

where $\:G$ denotes the graphene-assisted entrance layer, $\:H$ and $\:L$ represent the high- and low-refractive-index layers, respectively, $\:N$ is the number of periods on each side of the cavity, and $\:D$ is the oral-tissue defect layer. The defect layer acts as the active sensing region. Any change in the refractive index of this layer modifies the optical path length of the cavity and consequently shifts the defect-mode resonance wavelength.

The periodic multilayer sections form a photonic band gap due to Bragg interference. When the defect layer is inserted between the two Bragg mirrors, a localized optical state appears inside the photonic band gap. This localized state produces a narrow resonance feature in the reflectance and transmittance spectra. The sensing mechanism is therefore based on tracking the spectral position of this defect-mode resonance as the refractive index of the oral-tissue cavity changes.

For a homogeneous layer $\:j$, the characteristic matrix for transverse electric (TE) polarization is expressed as^13,14,21,22^

1 $$\:{M}_{j}=\left[\begin{array}{cc}\mathrm{c}\mathrm{o}\mathrm{s}{\delta\:}_{j}&\:\frac{i}{{\eta\:}_{j}}\mathrm{s}\mathrm{i}\mathrm{n}{\delta\:}_{j}\\\:i{\eta\:}_{j}\mathrm{s}\mathrm{i}\mathrm{n}{\delta\:}_{j}&\:\mathrm{c}\mathrm{o}\mathrm{s}{\delta\:}_{j}\end{array}\right].$$

where $\:{\delta\:}_{j}$ is the optical phase thickness and $\:{\eta\:}_{j}$ is the optical admittance of the $\:j$-th layer. The optical phase thickness is defined by

2 $$\:{\delta\:}_{j}=\frac{2\pi\:}{\lambda\:}{n}_{j}{d}_{j}\mathrm{c}\mathrm{o}\mathrm{s}{\theta\:}_{j}.$$

where $\:{n}_{j}$, $\:{d}_{j}$, and $\:{\theta\:}_{j}$ are the refractive index, thickness, and internal propagation angle of the $\:j$-th layer, respectively, and $\:\lambda\:$ is the operating wavelength. The propagation angle inside each layer is obtained from Snell’s law:

3 $$\:{n}_{0}\mathrm{s}\mathrm{i}\mathrm{n}{\theta\:}_{0}={n}_{j}\mathrm{s}\mathrm{i}\mathrm{n}{\theta\:}_{j}.$$

where $\:{n}_{0}$ and $\:{\theta\:}_{0}$ are the refractive index and incident angle of the input medium. For TE polarization, the optical admittance is given by

4 $$\:{\eta\:}_{j}={n}_{j}\mathrm{c}\mathrm{o}\mathrm{s}{\theta\:}_{j}.$$

The total transfer matrix of the complete multilayer sensor is obtained by multiplying the matrices of all layers in their physical order:

5 $$\:M=\prod\:_{j=1}^{{M}_{L}}{M}_{j}.$$

where $\:{M}_{L}$ is the total number of layers in the structure. For the proposed design, the total matrix includes the graphene-assisted entrance layer, the left periodic multilayer section, the oral-tissue defect cavity, the right periodic multilayer section, and the substrate boundary. The total matrix can be written as

6 $$\:M=\left[\begin{array}{cc}A&\:B\\\:C&\:D\end{array}\right].$$

The complex reflection coefficient is calculated from

7 $$\:r=\frac{{\eta\:}_{0}A+{\eta\:}_{0}{\eta\:}_{s}B-C-{\eta\:}_{s}D}{{\eta\:}_{0}A+{\eta\:}_{0}{\eta\:}_{s}B+C+{\eta\:}_{s}D}.$$

where $\:{\eta\:}_{0}$ and $\:{\eta\:}_{s}$ are the optical admittances of the incident and substrate media, respectively. The reflectance is therefore

8 $$\:R={\left|r\right|}^{2}.$$

In addition to the reflectance response, the transmittance spectrum is calculated to provide a complementary confirmation of the defect-mode resonance. The complex transmission coefficient is expressed as

9 $$\:t=\frac{2{\eta\:}_{0}}{{\eta\:}_{0}A+{\eta\:}_{0}{\eta\:}_{s}B+C+{\eta\:}_{s}D}.$$

and the transmittance is calculated as

10 $$\:T=\frac{\mathrm{R}\mathrm{e}\left({\eta\:}_{s}\right)}{\mathrm{R}\mathrm{e}\left({\eta\:}_{0}\right)}{\left|t\right|}^{2}.$$

The addition of the transmittance spectrum is important because the defect resonance appears as a transmission peak corresponding to the reflectance dip. Thus, reflectance and transmittance provide mutually consistent evidence for the existence and spectral shift of the localized defect mode.

The approximate resonance condition of the defect cavity can be described by the optical path relation

11 $$\:m{\lambda\:}_{\mathrm{r}\mathrm{e}\mathrm{s}}=2{n}_{D}{d}_{D}.$$

where $\:m$ is the resonance order, $\:{n}_{D}$ is the refractive index of the defect layer, $\:{d}_{D}$ is the defect-layer thickness, and $\:{\lambda\:}_{\mathrm{r}\mathrm{e}\mathrm{s}}$ is the resonance wavelength. This equation explains the physical origin of the observed red shift. When the refractive index of the oral-tissue defect layer increases, the optical path length of the cavity also increases. Consequently, the resonance condition is satisfied at a longer wavelength, which is the main reason for the systematic resonance displacement observed in the reflectance and transmittance spectra.

The resonance wavelength is extracted as the wavelength corresponding to the minimum reflectance inside the defect-mode search window:

12 $$\:{\lambda\:}_{\mathrm{r}\mathrm{e}\mathrm{s}}=\mathrm{a}\mathrm{r}\mathrm{g}\underset{\lambda\:}{\mathrm{m}\mathrm{i}\mathrm{n}}\hspace{0.17em}R\left(\lambda\:\right).$$

where the corresponding minimum reflectance is denoted by $\:{R}_{\mathrm{m}\mathrm{i}\mathrm{n}}$. The resonance dip depth is calculated as

13 $$\:{D}_{p}=1-{R}_{\mathrm{m}\mathrm{i}\mathrm{n}}.$$

A larger dip depth indicates a more visible resonance feature and improves the reliability of spectral readout.

The refractive-index sensitivity of the sensor is defined as

14 $$\:S=\frac{\varDelta\:{\lambda\:}_{\mathrm{r}\mathrm{e}\mathrm{s}}}{\varDelta\:{n}_{D}}.$$

where $\:\varDelta\:{\lambda\:}_{\mathrm{r}\mathrm{e}\mathrm{s}}$ is the resonance wavelength shift caused by a refractive-index change $\:\varDelta\:{n}_{D}$ in the defect cavity. In the present work, the global sensitivity is extracted from the slope of the linear calibration curve:

15 $$\:{\lambda\:}_{\mathrm{r}\mathrm{e}\mathrm{s}}=S{n}_{D}+b.$$

where $\:b$ is the intercept. This approach is more reliable than using isolated adjacent points because it evaluates the complete refractive-index range and reduces the influence of numerical wavelength discretization.

The linearity of the sensor response is evaluated using the coefficient of determination:

16 $$\:{R}^{2}=1-\frac{\sum\:_{i}{\left({\lambda\:}_{\mathrm{r}\mathrm{e}\mathrm{s},i}-{\lambda\:}_{\mathrm{f}\mathrm{i}\mathrm{t},i}\right)}^{2}}{\sum\:_{i}{\left({\lambda\:}_{\mathrm{r}\mathrm{e}\mathrm{s},i}-{\overline{\lambda\:}}_{\mathrm{r}\mathrm{e}\mathrm{s}}\right)}^{2}}.$$

where $\:{\lambda\:}_{\mathrm{r}\mathrm{e}\mathrm{s},i}$ are the extracted resonance wavelengths, $\:{\lambda\:}_{\mathrm{f}\mathrm{i}\mathrm{t},i}$ are the fitted values obtained from the calibration curve, and $\:{\overline{\lambda\:}}_{\mathrm{r}\mathrm{e}\mathrm{s}}$ is the mean resonance wavelength. A value of $\:{R}^{2}$ close to unity confirms a stable and predictable calibration response.

The full width at half maximum (FWHM) is used to evaluate the linewidth of the resonance dip. The half-depth reflectance level is calculated as

17 $$\:{R}_{\mathrm{h}\mathrm{a}\mathrm{l}\mathrm{f}}={R}_{\mathrm{m}\mathrm{i}\mathrm{n}}+\frac{1}{2}\left({R}_{b}-{R}_{\mathrm{m}\mathrm{i}\mathrm{n}}\right).$$

where $\:{R}_{b}$ is the local baseline reflectance around the resonance dip. The FWHM is then obtained from

18 $$\:\mathrm{FWHM}={\lambda\:}_{R}-{\lambda\:}_{L}.$$

where $\:{\lambda\:}_{L}$ and $\:{\lambda\:}_{R}$ are the left and right wavelengths at which the reflectance curve crosses the half-depth level. A smaller FWHM indicates a sharper resonance and better spectral discrimination.

The quality factor is calculated as

19 $$\:Q=\frac{{\lambda\:}_{\mathrm{r}\mathrm{e}\mathrm{s}}}{\mathrm{FWHM}}.$$

A high $\:Q$-factor means that the defect resonance is narrow and well confined. In sensing applications, this is important because a narrow resonance allows small wavelength shifts to be resolved more clearly.

The figure of merit combines sensitivity and resonance linewidth into a single performance parameter:

20 $$\:\mathrm{FOM}=\frac{S}{\mathrm{FWHM}}.$$

This metric is particularly useful because high sensitivity alone is not sufficient for reliable sensing. A sensor must provide both a large resonance shift and a narrow spectral linewidth. Therefore, the FOM gives a more balanced evaluation of the sensor performance.

The detection accuracy is defined as

21 $$\:\mathrm{DA}=\frac{1}{\mathrm{FWHM}}.$$

while the limit of detection is estimated as

22 $$\:\mathrm{LOD}=\frac{\mathrm{FWHM}}{S}.$$

This parameter represents the minimum refractive-index variation that can be resolved by the proposed sensor under the adopted spectral linewidth approximation. A lower LOD indicates better detection capability.

The electric-field distribution is calculated to verify the physical localization of the defect mode. The normalized field intensity is expressed as

23 $$\:I\left(z\right)={\left|E\left(z\right)\right|}^{2}=E\left(z\right)\overline{E\left(z\right)}.$$

where $\:E\left(z\right)$ is the electric-field amplitude along the propagation direction inside the multilayer stack and $\:\overline{E\left(z\right)}$ is its complex conjugate. The normalized intensity identifies the spatial region in which the optical field is concentrated. Strong localization inside the defect layer confirms that the sensing response is dominated by light-matter interaction inside the analyte-filled cavity rather than by weak distributed interaction across the whole multilayer structure.

This field-localization analysis is essential for explaining the physical origin of the high sensitivity. When the electromagnetic field is concentrated inside the defect cavity, small refractive-index variations in the oral-tissue layer strongly perturb the localized optical mode. This perturbation produces a measurable shift in $\:{\lambda\:}_{\mathrm{r}\mathrm{e}\mathrm{s}}$. Therefore, the calculated sensitivity is not only a numerical fitting result but is physically supported by the defect-mode confinement shown in the field-distribution profiles.

The dependence of the sensor response on incident angle is also investigated. According to Eq. (2), changing the incidence angle modifies the optical phase accumulated inside each layer. Consequently, the spectral position of the defect mode shifts with angle. The angle-wavelength reflectance map is therefore included to evaluate whether the resonance remains visible under oblique illumination. This is relevant because practical optical interrogation may involve small angular deviations from normal incidence. The persistence of the resonance under angular variation confirms that the sensor response remains physically stable.

The influence of defect-layer thickness is examined because $\:{d}_{D}$ directly controls the cavity optical path length. From Eq. (10), changing $\:{d}_{D}$ changes the resonance wavelength and affects the sensing response. Therefore, the sensitivity as a function of defect-layer thickness is evaluated using the fitted sensitivity extracted at different $\:{d}_{D}$ values:

24 $$\:S\left({d}_{D}\right)=\frac{\varDelta\:{\lambda\:}_{\mathrm{r}\mathrm{e}\mathrm{s}}\left({d}_{D}\right)}{\varDelta\:{n}_{D}}.$$

This analysis identifies the role of defect thickness in improving or degrading sensor performance. Increasing the defect thickness increases the interaction length inside the analyte cavity, which can enhance the resonance shift. However, excessive thickness may broaden or destabilize the resonance. Therefore, the selected cavity thickness must balance sensitivity, Q-factor, and spectral stability.

The structural optimization is further supported by analyzing the quality factor as a function of both the number of periods and defect thickness:

25 $$\:Q\left(N,{d}_{D}\right)=\frac{{\lambda\:}_{\mathrm{r}\mathrm{e}\mathrm{s}}\left(N,{d}_{D}\right)}{\mathrm{FWHM}\left(N,{d}_{D}\right)}.$$

Increasing the number of periods strengthens the Bragg mirrors surrounding the defect cavity, enhances optical confinement, and generally sharpens the resonance. However, very large numbers of periods may increase fabrication complexity. Thus, the selected structure represents a compromise between optical performance and practical design simplicity.

To evaluate fabrication robustness, a uniform thickness deviation is introduced to all layer thicknesses:

26 $$\:{d}_{j}{\prime\:}={d}_{j}\left(1+\epsilon\right).$$

where $\:{d}_{j}{\prime\:}$ is the perturbed thickness and $\:\epsilon$ is the relative thickness deviation. The main sensing parameters are recalculated under different thickness deviations. This tolerance analysis evaluates the stability of the sensitivity, Q-factor, FWHM, and LOD under possible fabrication imperfections. A stable response under small thickness deviations indicates that the proposed structure is not overly sensitive to minor fabrication errors.

The proposed model is theoretical and assumes an effective refractive-index representation of healthy and cancerous oral-tissue states. The analyte is assumed to fill or effectively occupy the defect cavity uniformly. This assumption is commonly used in transfer-matrix-based refractive-index sensing models to isolate the optical response of the sensor to controlled changes in the analyte refractive index. In practical implementation, this condition can be approached by using a thin oral-fluid or tissue-derived sample layer deposited into or placed in contact with the sensing cavity. Therefore, the present analysis should be considered a proof-of-concept optical design that requires future experimental validation.

The reflectance dip and transmittance peak confirm the presence of a localized defect resonance. The calibration curve quantifies the refractive-index sensitivity. The Q-factor, FWHM, FOM, DA, LOD, and dip depth provide a complete evaluation of resonance quality and detection capability. The field-distribution maps demonstrate that the optical mode is strongly localized inside the analyte cavity. The angular, thickness, and tolerance analyses further confirm that the proposed design provides a physically consistent and robust platform for oral cancer-related refractive-index sensing.

## Results and discussion

**Fig. 1:**
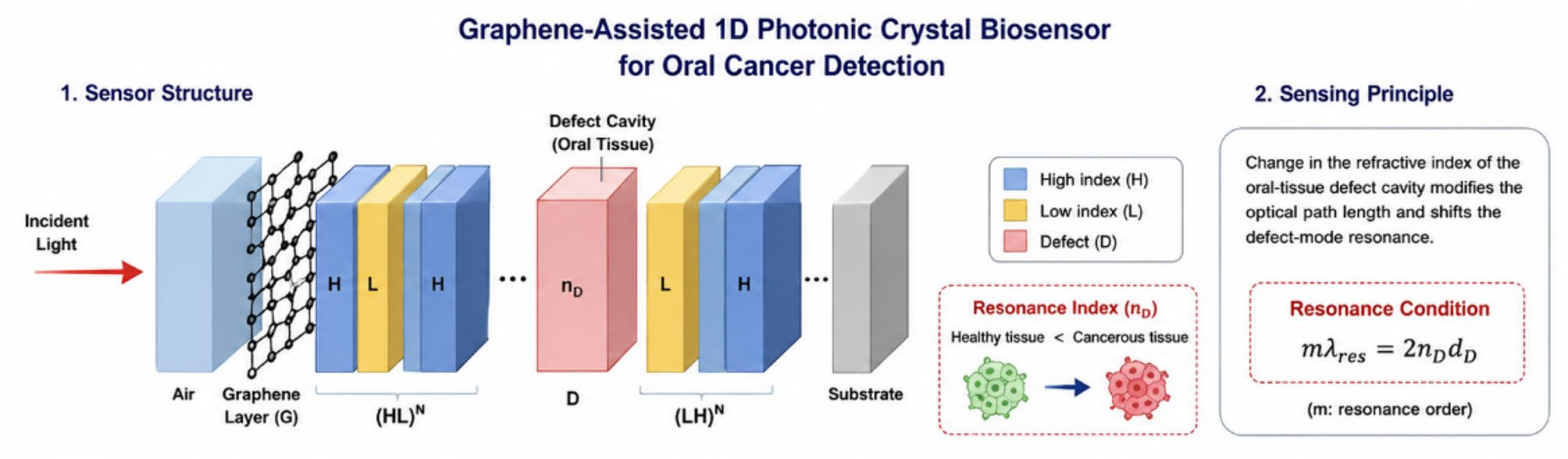
Simplified schematic illustration of the proposed graphene-assisted one-dimensional photonic crystal biosensor for oral cancer detection. Variations in the refractive index of the oral-tissue defect cavity induce measurable shifts in the localized defect-mode resonance inside the photonic band gap, enabling sensitive discrimination between healthy and cancerous tissues.

Figure 1 schematically presents the design concept and sensing mechanism of the proposed graphene-assisted 1D photonic crystal biosensor for oral cancer detection. The structure consists of alternating high- and low-refractive-index layers surrounding an oral-tissue defect cavity. This cavity supports a localized defect-mode resonance inside the photonic band gap. When the refractive index changes from healthy to cancerous tissue values, the optical path length of the cavity changes, leading to a measurable resonance shift according to mλres=2nDdD. This mechanism is the basis for the reflectance/transmittance response, field localization, calibration behavior, and sensing performance discussed in the following figures. The multilayer configuration supports optical confinement near the defect-mode resonance and enhances the localized interaction between the optical field and the oral-tissue defect cavity.

**Fig. 2:**
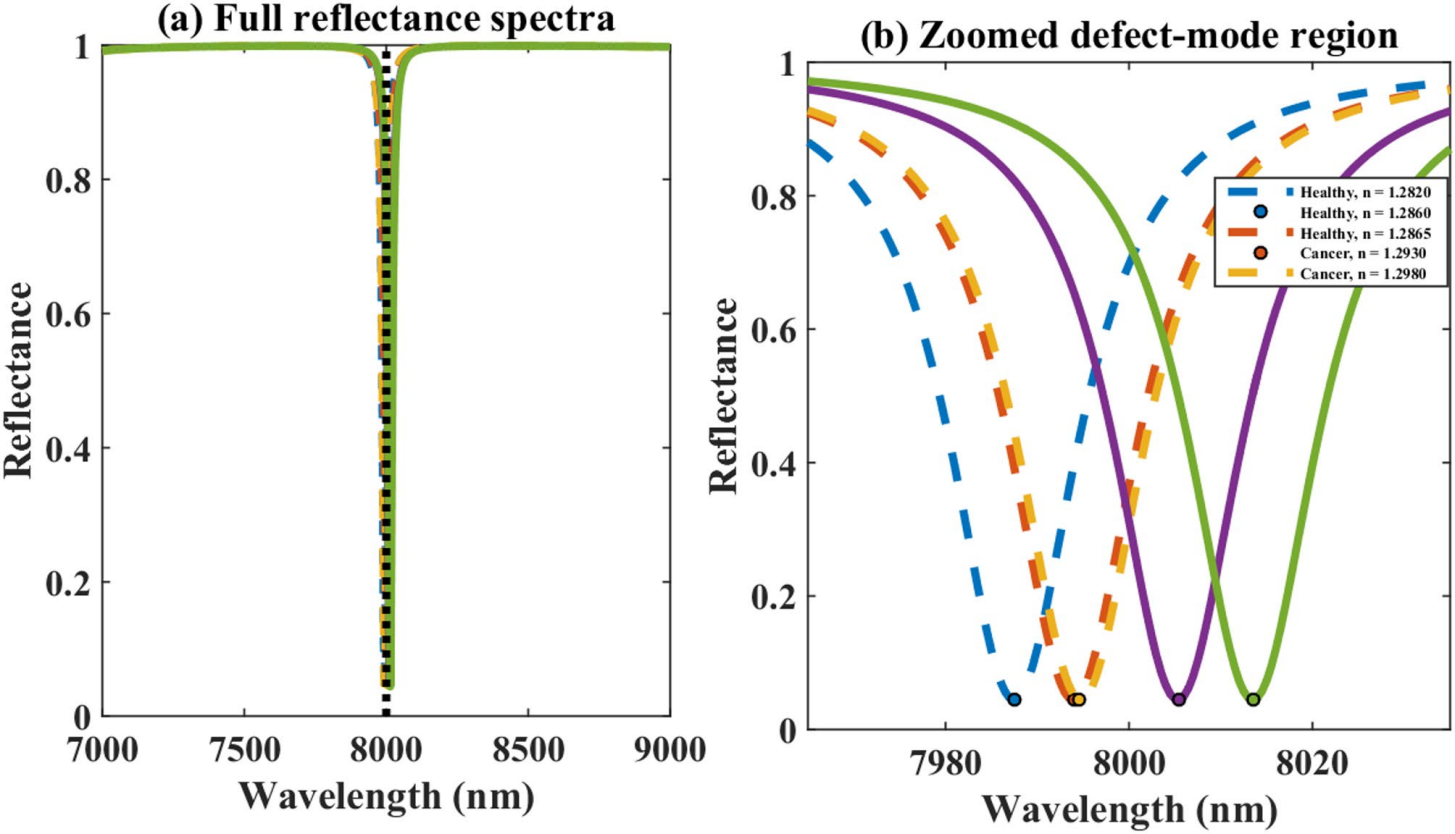
(**a**) Full reflectance spectra of the proposed photonic crystal biosensor. (**b**) Enlarged defect-mode resonance region showing the red shift of the resonance wavelength with increasing oral-tissue refractive index.

Figure 2(a) presents the full reflectance spectra of the proposed structure over the investigated mid-infrared wavelength range. A pronounced photonic band gap with near-unity reflectance is observed, confirming the strong Bragg interference generated by the periodic multilayer arrangement. Inside this forbidden spectral region, a sharp defect-mode resonance dip appears near 8000 nm. The existence of this narrow resonance demonstrates the formation of a strongly localized optical state inside the oral-tissue cavity.

The enlarged resonance region shown in Fig. 2(b) clearly demonstrates the systematic red shift of the defect resonance as the refractive index of the analyte cavity increases from healthy to cancerous tissue values. This behavior originates from the increase in the optical path length inside the defect cavity according to the resonance condition:

$$\:\boldsymbol{m}{\boldsymbol{\lambda\:}}_{\boldsymbol{r}\boldsymbol{e}\boldsymbol{s}}=2{\boldsymbol{n}}_{\boldsymbol{D}}{\boldsymbol{D}}_{\boldsymbol{D}}$$

As the refractive index increases, the phase accumulation inside the cavity becomes larger, and consequently the resonance condition is satisfied at longer wavelengths. The resonance dip remains extremely narrow throughout the investigated refractive-index range, indicating that the cavity preserves strong optical confinement and high spectral selectivity. Moreover, the resonance depth remains almost unchanged, indicating stable energy localization and low resonance degradation under refractive-index variation. The observed resonance shifts verify that the proposed sensor can distinguish healthy and cancerous oral tissue states through optical spectral interrogation. The narrow linewidth additionally suggests strong cavity confinement and improved spectral resolution.

**Fig. 3:**
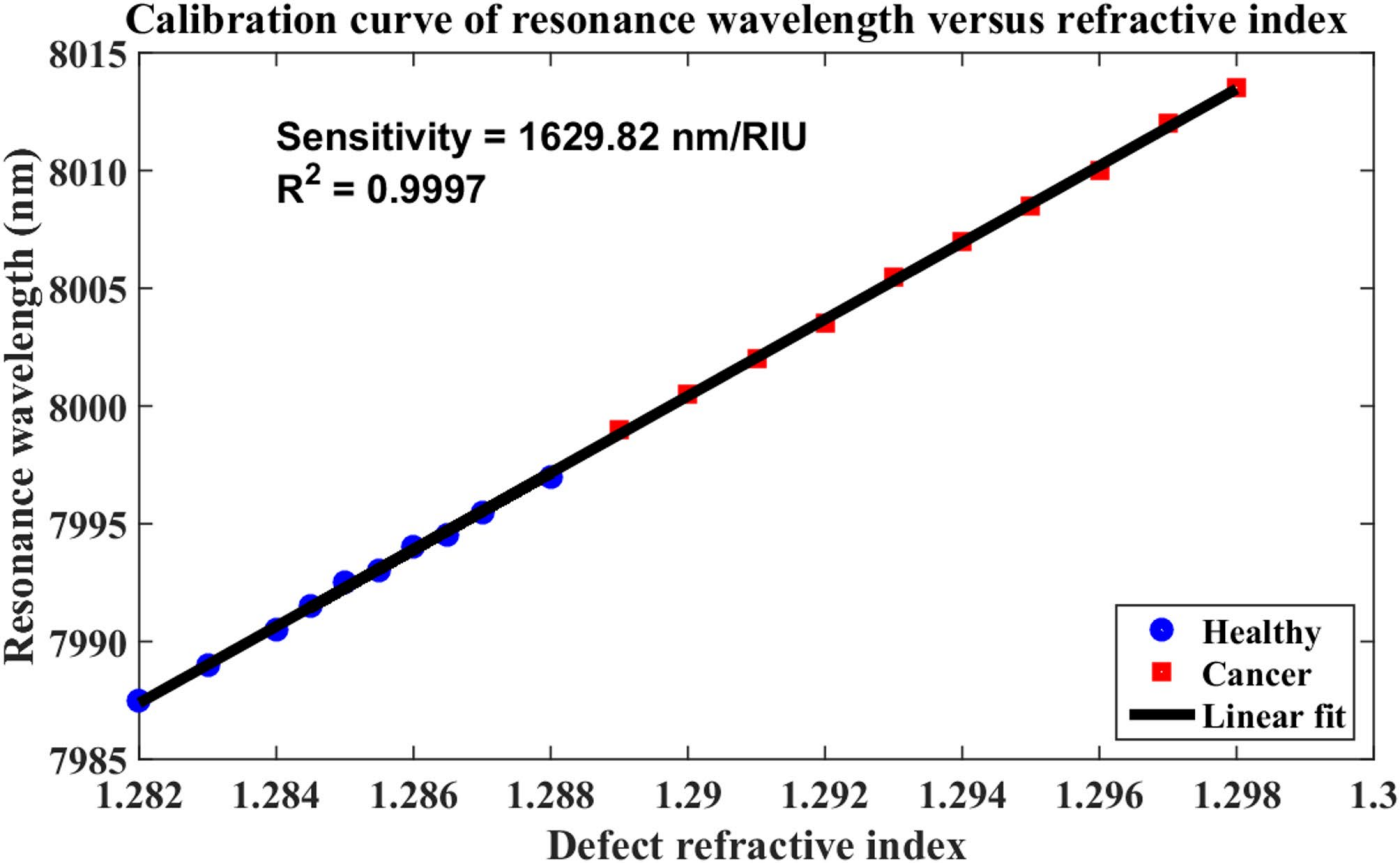
Calibration curve of resonance wavelength versus oral-tissue refractive index showing highly linear sensing behavior.

Figure 3 presents the calibration curve relating the resonance wavelength to the refractive index of the oral-tissue defect cavity. A highly linear relationship is obtained with a sensitivity of approximately 1629.82 nm/RIU and a coefficient of determination R^2^ = 0.9997. The excellent linearity confirms that the resonance shift is directly governed by the cavity refractive index without significant nonlinear spectral distortion.

Physically, the linearity arises because the defect resonance depends predominantly on the effective optical path length inside the analyte cavity. Since the refractive-index variations remain relatively small within the investigated biological range, the resonance perturbation behaves approximately linearly. This is highly desirable for biosensing applications because it simplifies calibration and improves measurement predictability. The large resonance displacement observed in Fig. 3 confirms strong interaction between the confined electromagnetic field and the analyte cavity. This behavior is directly associated with the localization of the defect mode inside the oral-tissue layer. Therefore, the proposed structure provides both high sensitivity and stable spectral behavior.

**Fig. 4:**
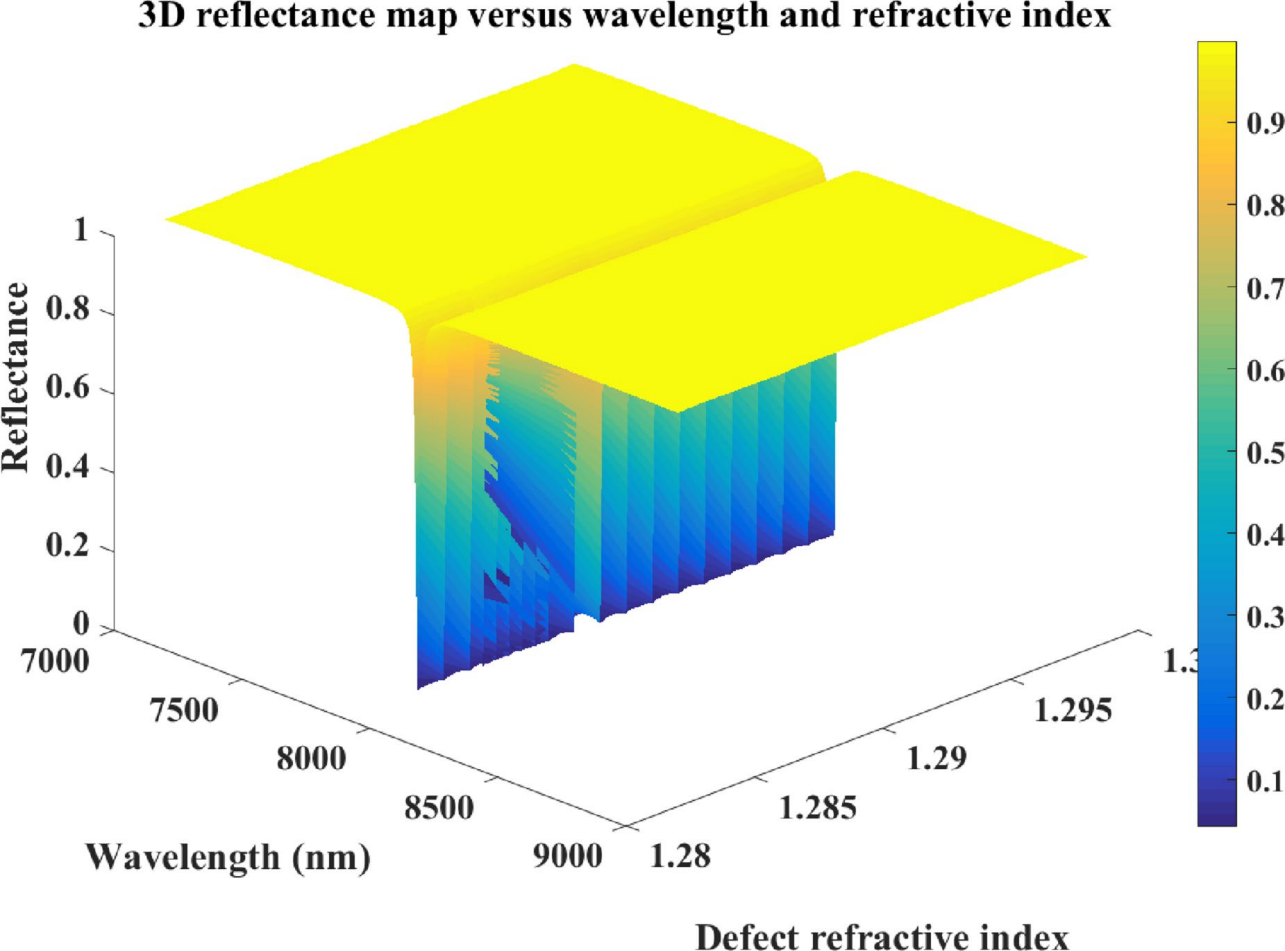
3D reflectance map versus wavelength and defect refractive index.

Figure 4 shows the three-dimensional reflectance map as a function of wavelength and refractive index. The defect resonance appears as a continuous low-reflectance valley inside the photonic band gap. The gradual displacement of this valley toward longer wavelengths confirms the systematic resonance shift induced by refractive-index variation.

This figure provides additional physical insight beyond the two-dimensional spectra. The smooth and continuous evolution of the resonance trajectory indicates stable cavity-mode formation without abrupt spectral distortion or mode splitting. The absence of discontinuities also confirms that the investigated refractive-index range does not induce instability in the defect mode.

Furthermore, the sharpness of the resonance valley demonstrates that theoptical energy remains strongly confined inside the cavity while leakageinto the surrounding Bragg mirrors remains limited. This strong localization is responsible for the high sensitivity achieved by the proposed design.

**Fig. 5:**
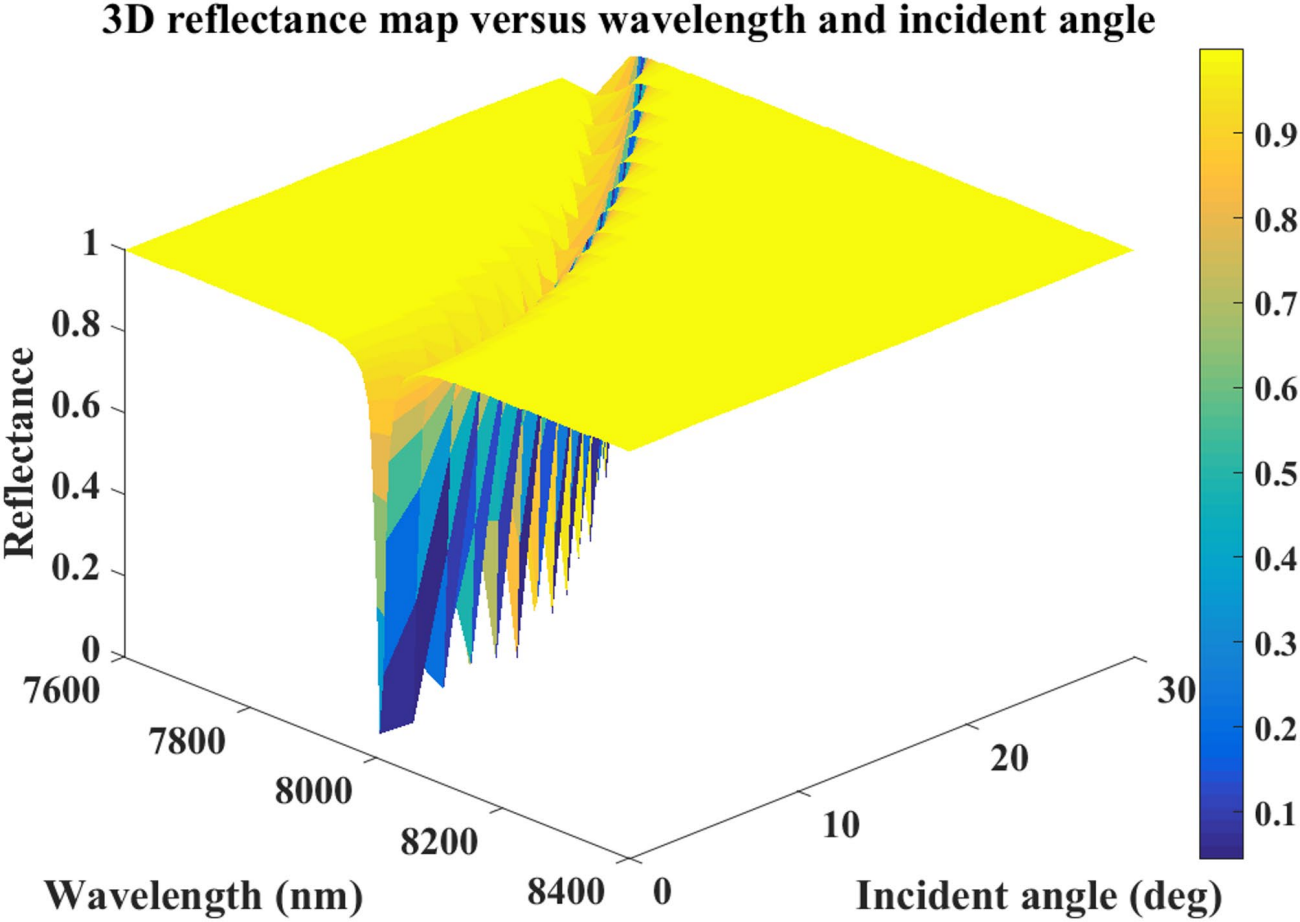
3D-reflectance map versus wavelength and incident angle.

The angle-dependent reflectance response confirms the angular behavior of the proposed structure, as shown in Fig. 5. With increasing incident angle, the resonance wavelength progressively shifts due to the change in the effective optical phase accumulated within each layer. This phase variation arises because the optical phase thickness depends on the propagation angle according to:

$$\:{\boldsymbol{\delta\:}}_{\boldsymbol{j}}=\frac{2\boldsymbol{\pi\:}}{\boldsymbol{\lambda\:}}{\boldsymbol{n}}_{\boldsymbol{j}}{\boldsymbol{d}}_{\boldsymbol{j}}\mathbf{c}\mathbf{o}\mathbf{s}{\boldsymbol{\theta\:}}_{\boldsymbol{j}}$$

As the incident angle changes, the optical path length inside the multilayer structure is modified, resulting in displacement of the resonance position. Despite this angular variation, the defect mode remains clearly observable throughout the investigated angular range, indicating strong resonance stability.

**Fig. 6:**
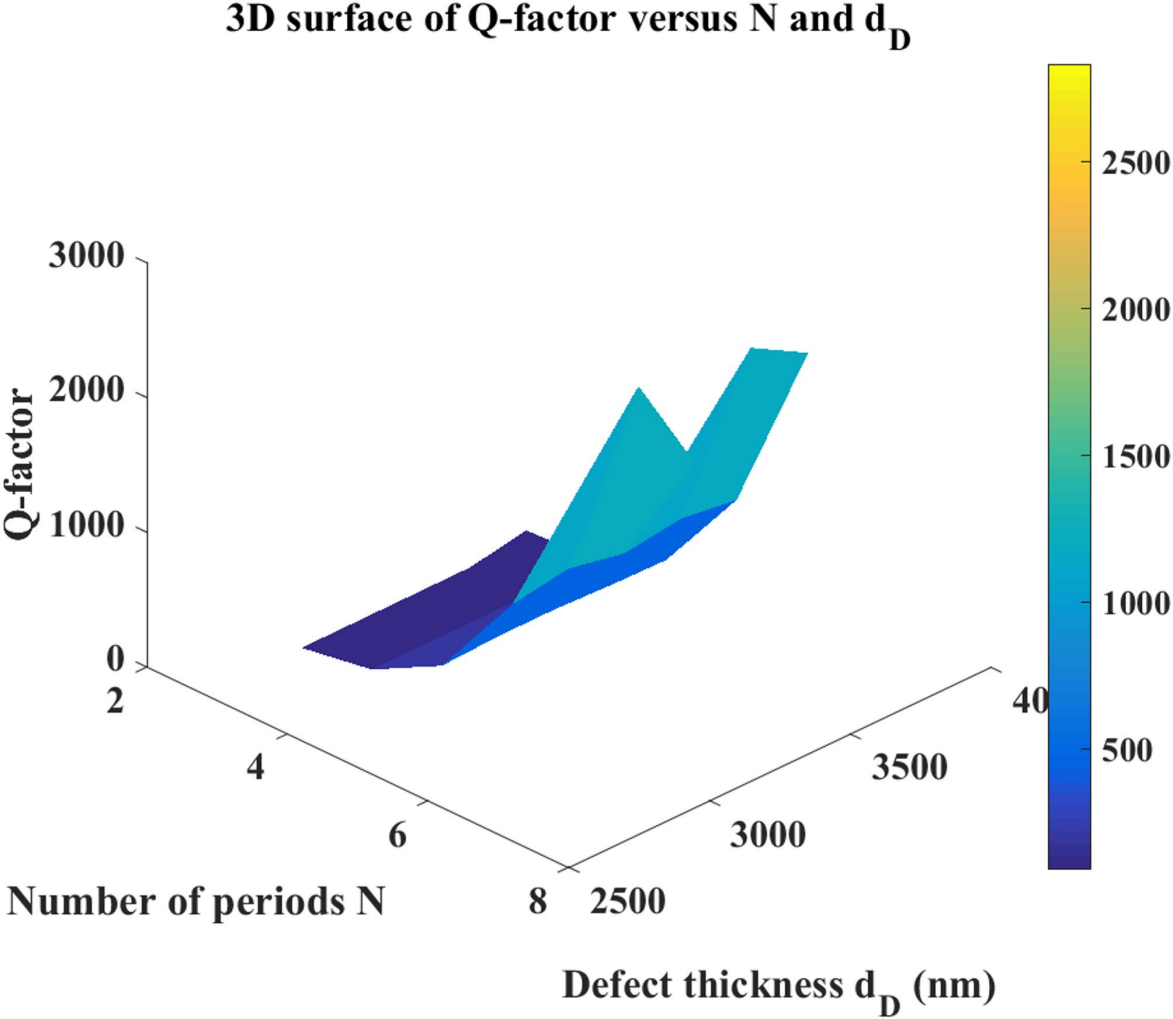
Three-dimensional surface of the Q-factor versus the number of periods and defect-layer thickness.

Figure 6 presents the dependence of the quality factor on both the number of periods and the defect-layer thickness. Increasing the number of periods enhances the Bragg reflectivity of the surrounding mirrors and therefore improves optical confinement inside the defect cavity. Consequently, the resonance linewidth becomes narrower and the Q-factor increases.

The defect thickness also plays an important role because it controls the optical path length and field localization inside the cavity. However, excessive defect thickness may broaden the resonance due to reduced confinement efficiency. Therefore, the optimal structure must balance resonance sharpness and field interaction length. The results in Fig. 6 confirm that the selected structural parameters provide a suitable compromise between optical confinement, sensitivity, and spectral stability.

**Fig. 7:**
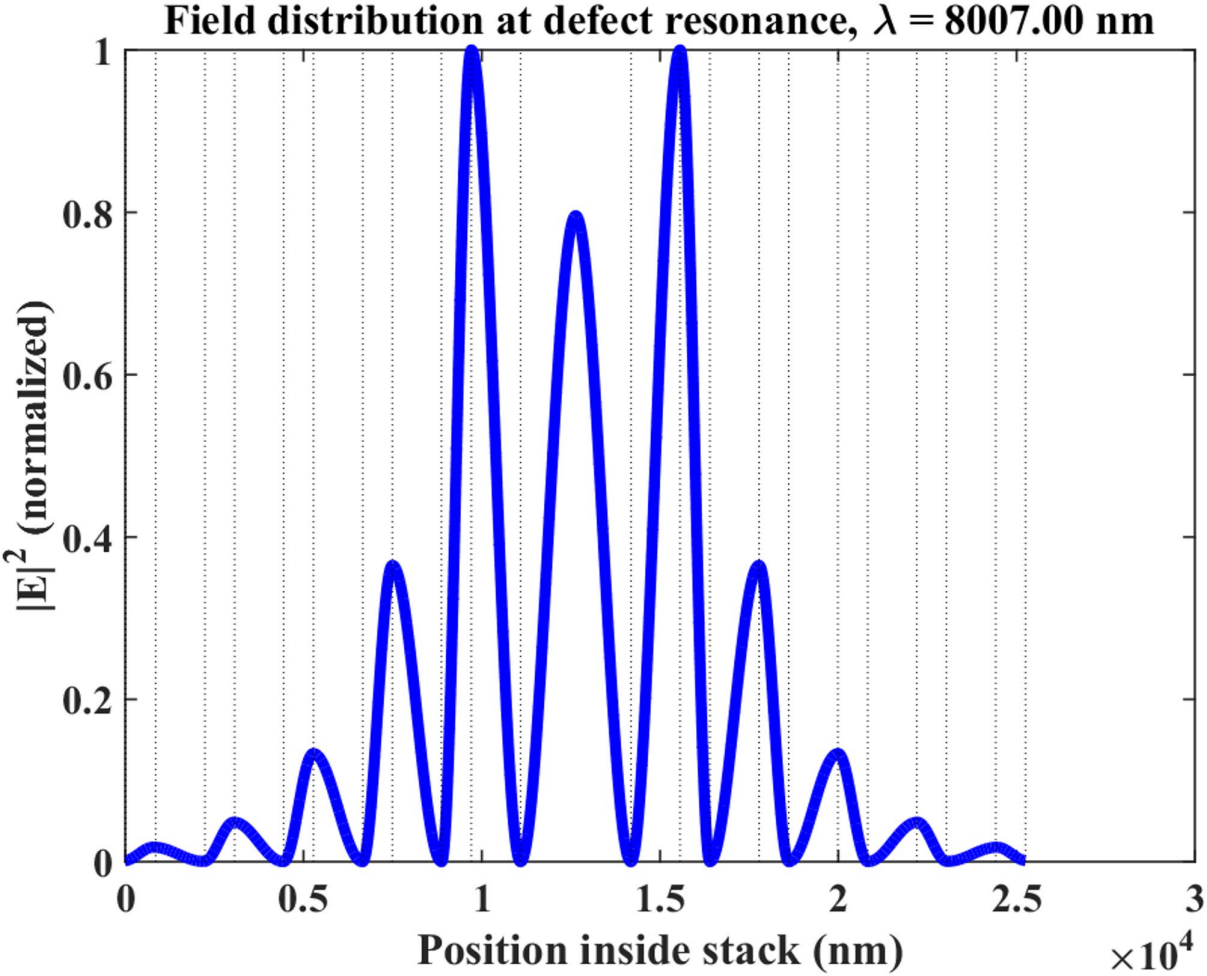
Normalized electric-field distribution inside the multilayer structure at the defect resonance wavelength.

Figure 7 shows the normalized electric-field distribution inside the multilayer stack at the defect resonance wavelength. Strong field localization is clearly observed around the central defect cavity, confirming the formation of a confined resonant mode. The localization of the electromagnetic field inside the analyte region is physically significant because it maximizes the overlap between the optical mode and the oral-tissue cavity. Consequently, even small refractive-index perturbations strongly influence the resonance condition, leading to measurable wavelength shifts. The field amplitude decays rapidly away from the defect region due to the strong Bragg reflection generated by the surrounding periodic multilayer structure. This confirms that the photonic crystal mirrors efficiently confine the optical energy inside the cavity and suppress radiation leakage. Therefore, the high sensitivity obtained in the proposed biosensor is not merely a numerical fitting result but is directly supported by strong electromagnetic localization inside the analyte cavity.

**Fig. 8:**
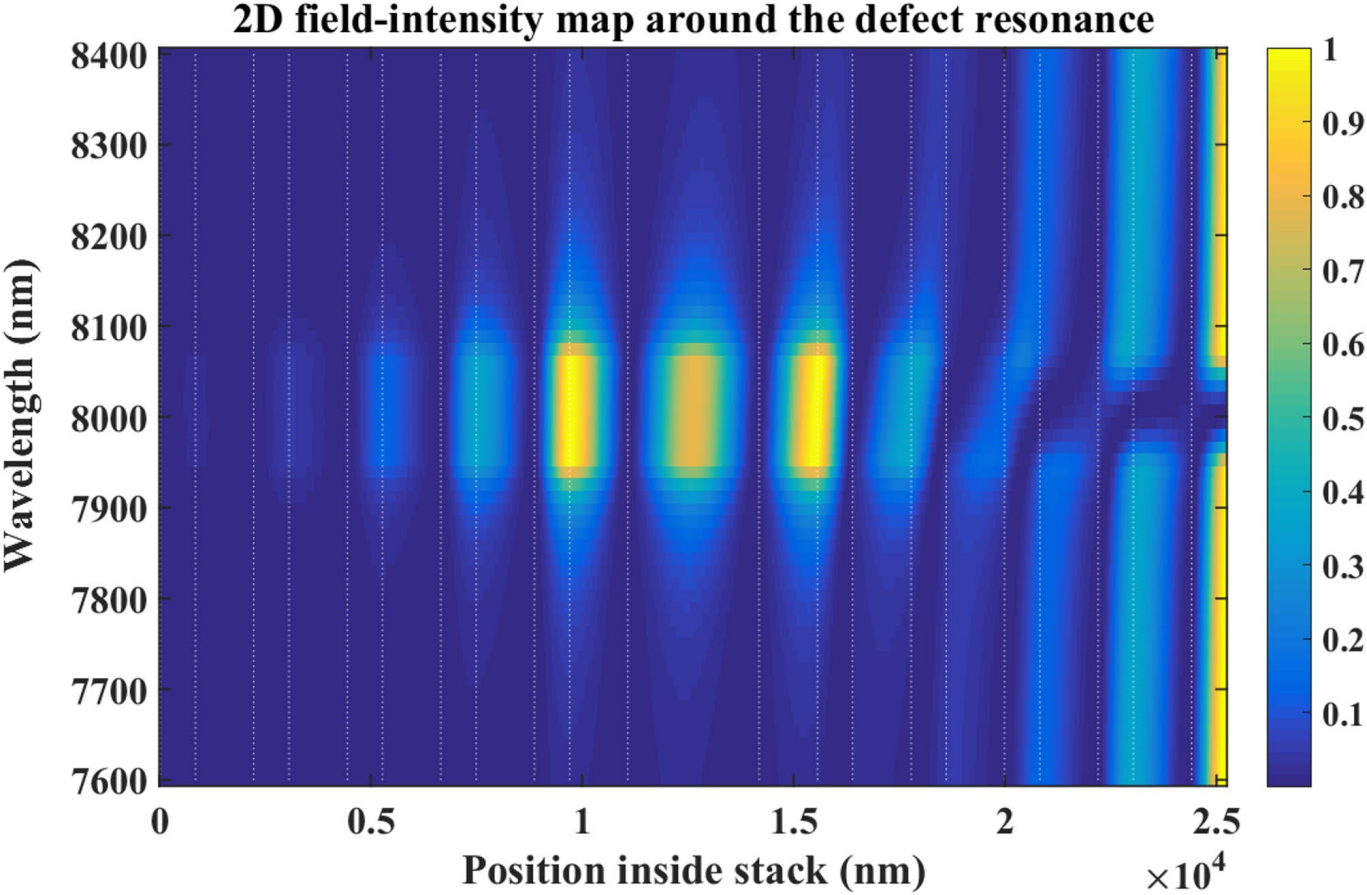
Two-dimensional electric-field intensity map around the defect resonance wavelength.

Figure 8 presents the two-dimensional electric-field intensity map around the resonance wavelength. The figure demonstrates strong spatial localization of the electromagnetic field near the defect region and spectral concentration around the resonance wavelength. The concentrated intensity region confirms that the defect resonance remains spatially and spectrally well defined. This dual confinement is essential for achieving strong light–matter interaction and high refractive-index sensitivity. Additionally, the narrow spectral localization observed in Fig. 8 is consistent with the high Q-factor and small FWHM obtained from the resonance spectra. Therefore, the field-intensity map provides physical validation of the sharp resonance behavior observed in the reflectance and transmittance responses.

**Fig. 9:**
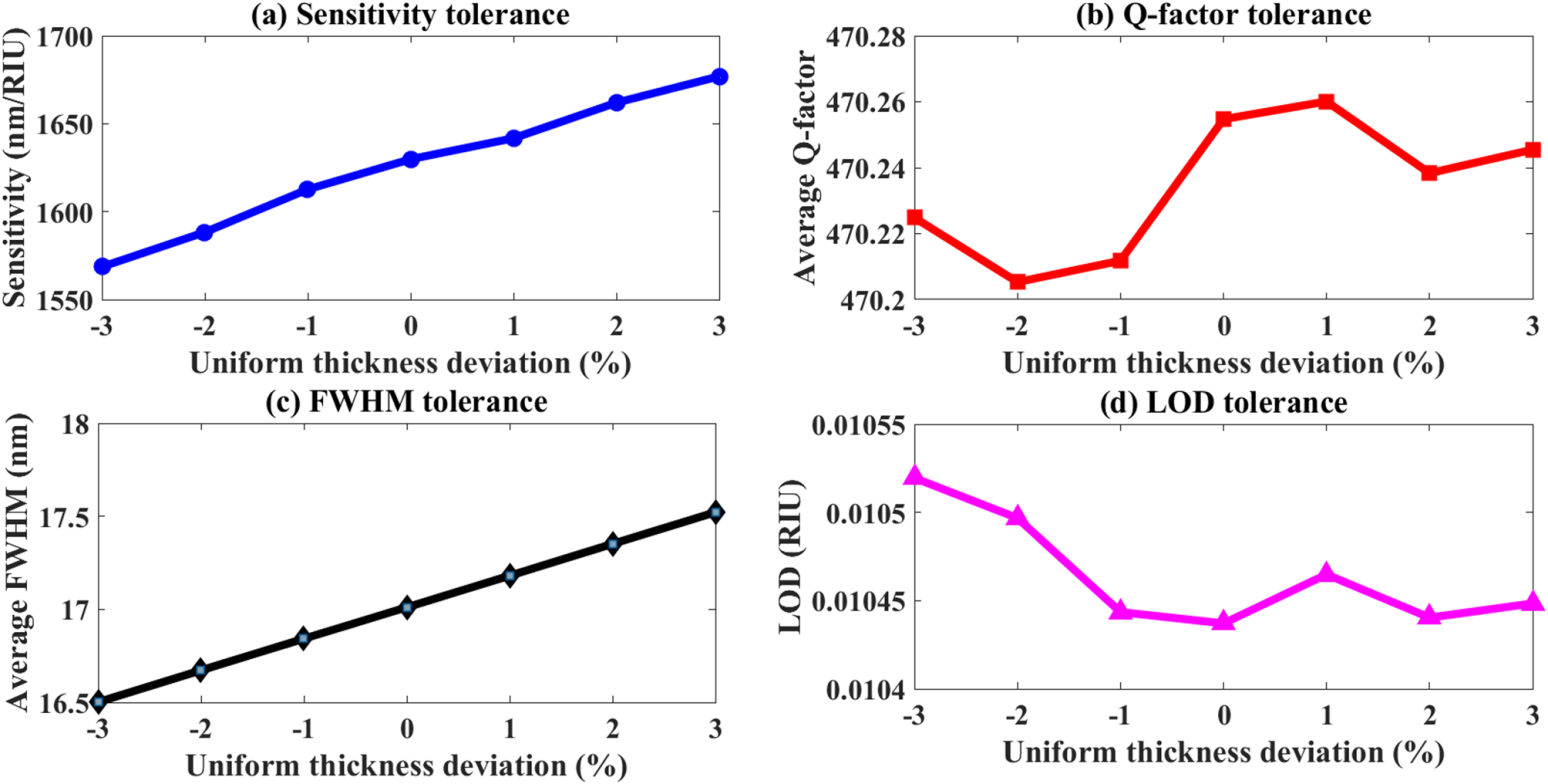
Tolerance analysis showing the effect of uniform thickness deviations on (**a**) sensitivity, (**b**) Q-factor, (**c**) FWHM, and (**d**) LOD.

Figure 9 illustrates the tolerance analysis of the proposed structure under uniform thickness deviations. The sensitivity, Q-factor, FWHM, and LOD remain relatively stable under small fabrication perturbations, demonstrating strong structural robustness.

The sensitivity variation remains limited even when thickness deviations are introduced, indicating that the resonance shift mechanism is not excessively sensitive to small fabrication errors. Similarly, the Q-factor exhibits only minor fluctuations, confirming that optical confinement remains largely preserved. The FWHM increases slightly under larger deviations, which is expected because fabrication imperfections perturb the phase matching inside the multilayer cavity and slightly weaken resonance confinement. These results indicate that the sensing response is not strongly degraded by small uniform thickness errors. This analysis provides a useful estimate of the expected response under non-ideal layer-thickness control.

**Fig. 10:**
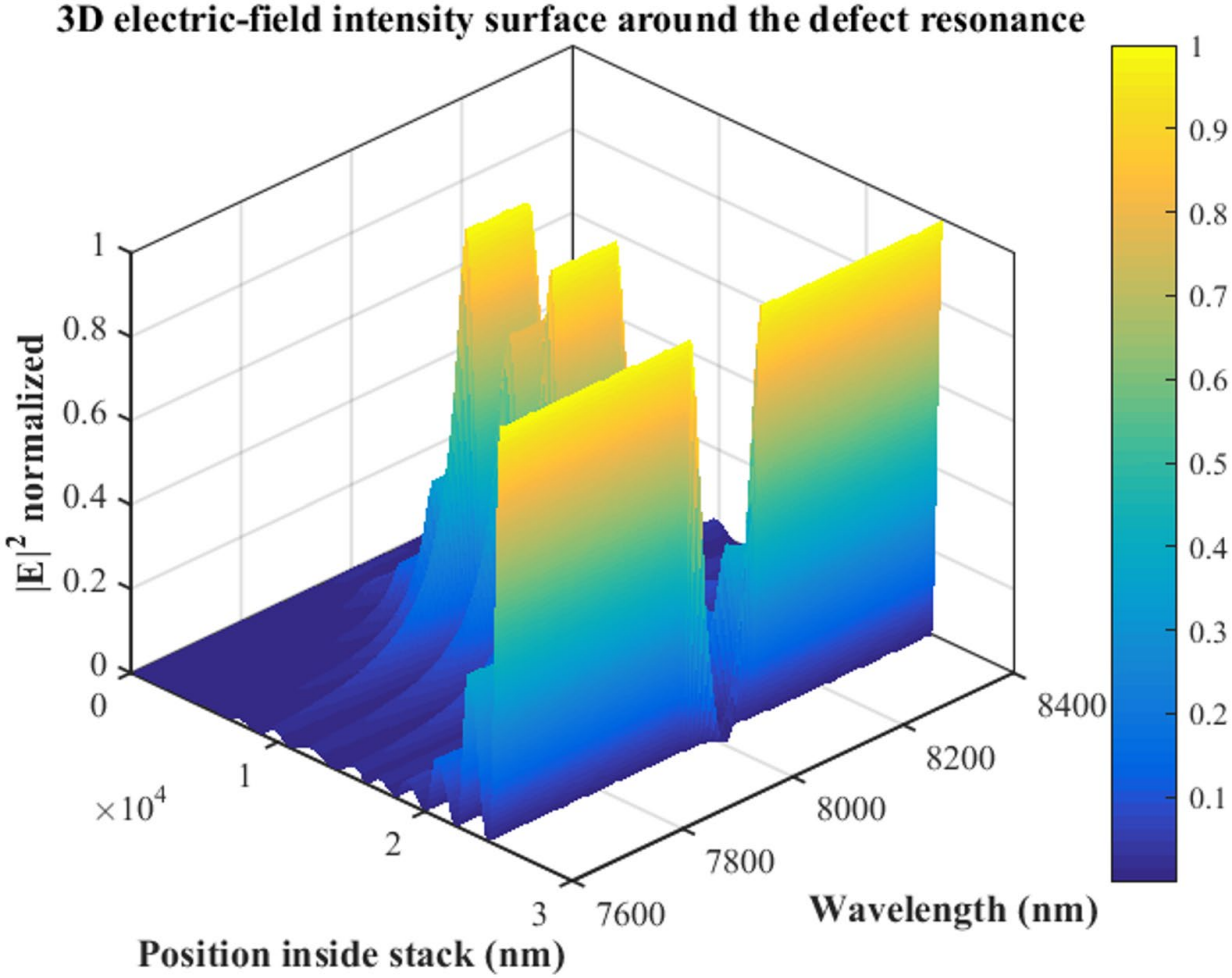
Three-dimensional electric-field intensity distribution around the defect resonance wavelength.

Figure 10 presents the three-dimensional electric-field intensity distribution around the defect resonance. The strong field enhancement inside the cavity region confirms efficient optical confinement and localized energy storage. The field distribution additionally reveals that the resonance energy is concentrated predominantly near the defect region while the surrounding multilayer sections act as highly reflective mirrors. This behavior is characteristic of defect-mode photonic crystal resonators.

Figure 10 further supports the cavity-based sensing mechanism by showing that the resonant energy is concentrated around the defect region.

**Fig. 11:**
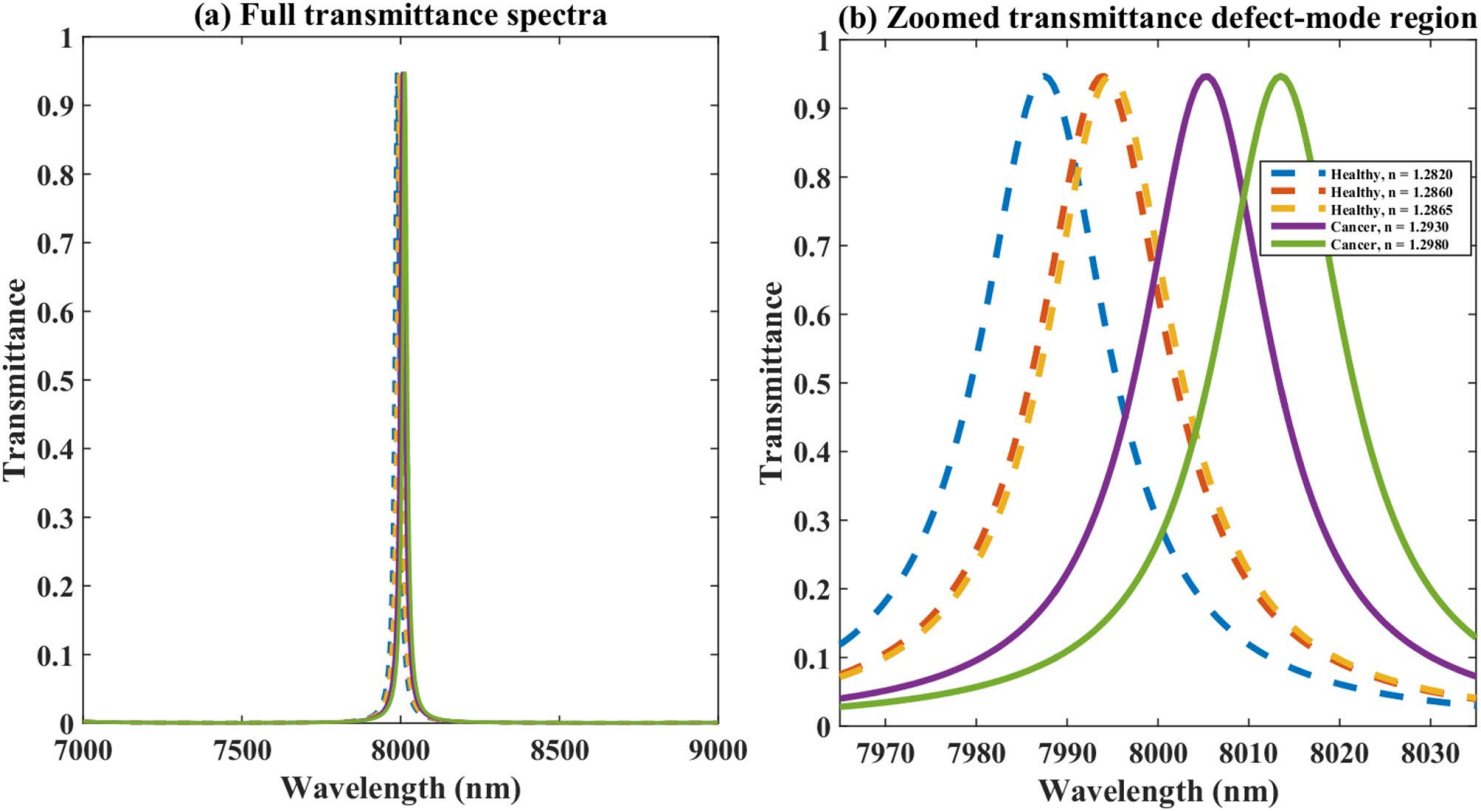
(**a**) Full transmittance spectra of the proposed structure. (**b**) Enlarged transmittance resonance region showing resonance displacement under refractive-index variation.

Figure 11 presents the transmittance spectra corresponding to the investigated reflectance responses. A sharp transmission peak appears inside the photonic band gap exactly at the defect resonance wavelength. This transmission peak corresponds to the localized defect mode and provides complementary confirmation of the resonance behavior. As the refractive index increases, the transmission resonance shifts toward longer wavelengths, consistent with the reflectance analysis. The simultaneous observation of a reflectance dip and a transmission peak confirms that the resonance originates from a genuine localized cavity mode rather than numerical artifacts. The narrow transmission linewidth additionally confirms strong optical confinement and low resonance leakage.

**Fig. 12:**
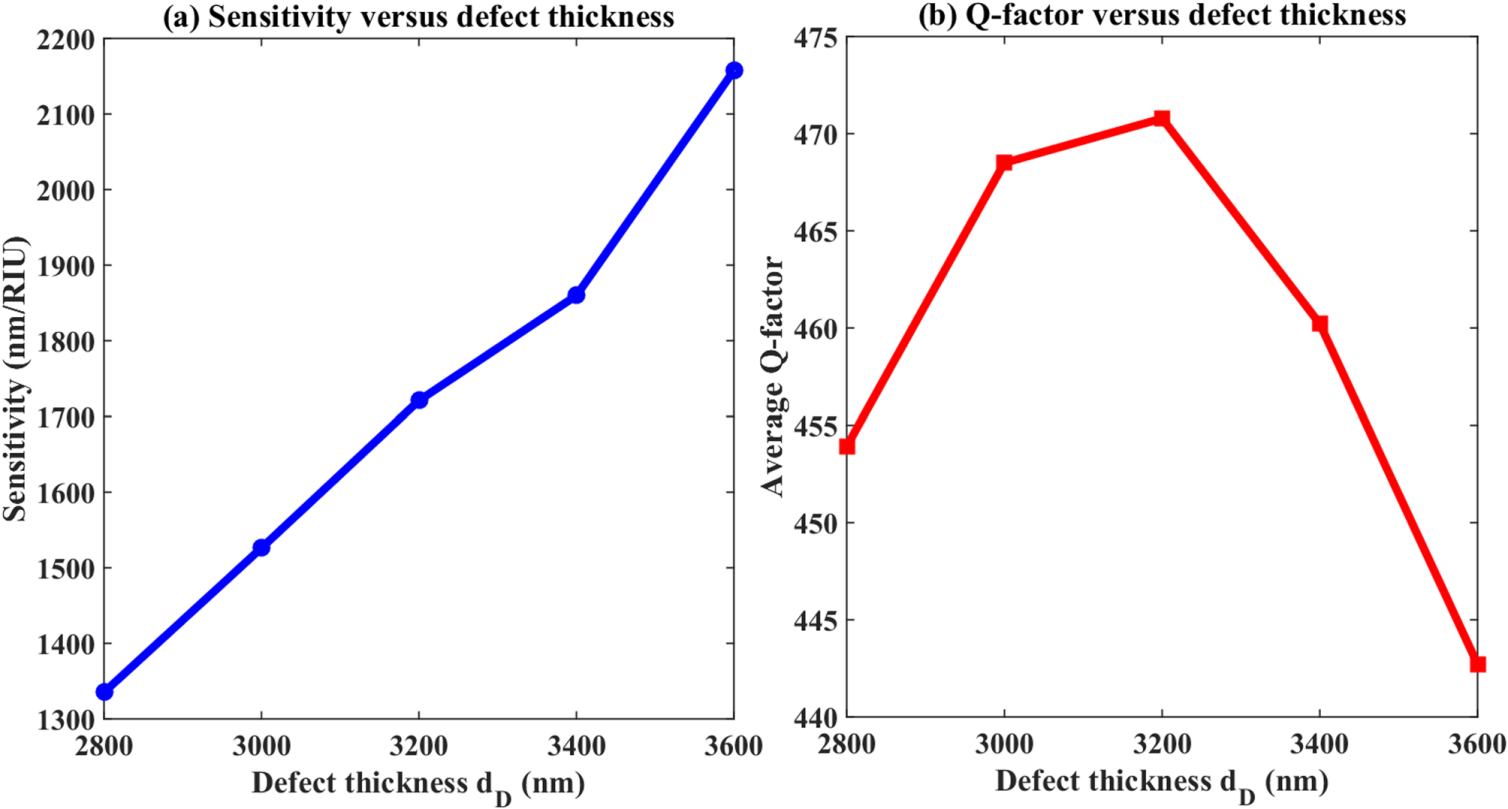
(**a**) Sensitivity versus defect-layer thickness. (**b**) Q-factor versus defect-layer thickness.

Figure 12 illustrates the effect of defect-layer thickness on both sensitivity and Q-factor. Increasing the defect thickness increases the interaction length between the optical field and the analyte cavity, which enhances the resonance shift and therefore increases sensitivity. However, the Q-factor does not continuously increase with thickness. After reaching an optimum region, the resonance confinement gradually weakens and the linewidth broadens, causing a reduction in the Q-factor. This behavior indicates that excessively large cavities reduce confinement efficiency despite increasing optical interaction length. Therefore, Fig. 12 demonstrates that an optimal defect thickness is necessary to simultaneously achieve strong sensitivity and high spectral quality.

The combined spectral, field-distribution, and tolerance results provide a consistent assessment of the proposed defect-mode sensing mechanism. The combination of narrow resonance linewidth, high Q-factor, strong field localization, and large resonance shifts confirms the suitability of the proposed structure for highly sensitive oral cancer detection in the mid-infrared spectral region.

**Table 1:** Key sensing parameters including sensitivity, resolution, and detection capability.

Parameter	Value
Sensitivity (nm/RIU)	1629.82
FWHM (nm)	17.01
Quality Factor (Q)	470.25
Figure of Merit (RIU⁻¹)	95.81
Detection Accuracy (nm⁻¹)	0.0588
Limit of Detection (RIU)	0.0104
Dip Depth	0.956

Table 1 summarizes the main sensing parameters extracted from the resonance analysis using the performance equations defined in the theoretical section. These parameters include the sensitivity, FWHM, Q-factor, figure of merit, detection accuracy, limit of detection, and dip depth. Physically, these metrics are important because they do not evaluate the sensor response from the resonance shift alone. Instead, they describe the combined effect of wavelength displacement, resonance sharpness, spectral visibility, and detection capability. The high sensitivity of 1629.82 nm/RIU confirms that the defect resonance is strongly affected by refractive-index variations in the oral-tissue cavity. The relatively narrow FWHM of 17.01 nm and Q-factor of 470.25 indicate that the localized defect mode remains spectrally sharp and well confined. In addition, the FOM of 95.81 RIU⁻¹, detection accuracy of 0.0588 nm⁻¹, LOD of 0.0104 RIU, and dip depth of 0.956 confirm that the resonance is sufficiently clear and measurable for refractive-index-based discrimination.

It should be noted that Table 2 compares sensors based on different physical principles, material systems, operating conditions, and readout methods. Therefore, the comparison should not be interpreted as a direct one-to-one ranking. For example, SPR sensors are often evaluated using angular sensitivity in °/RIU, whereas photonic crystal sensors are commonly evaluated using wavelength sensitivity in nm/RIU. This difference limits direct numerical comparison between all listed structures. Accordingly, the purpose of Table 2 is to provide a general performance context and to position the proposed sensor among related optical biosensing approaches.

Within this context, the proposed graphene-assisted one-dimensional photonic crystal sensor shows a balanced response for oral-cancer-related refractive-index detection. Compared with some previously reported photonic crystal sensors, the proposed structure combines high wavelength sensitivity with a reasonably high Q-factor and clear defect-mode resonance. More importantly, the present work evaluates the sensor using multiple physically meaningful parameters rather than sensitivity alone. The inclusion of FWHM, Q-factor, FOM, detection accuracy, LOD, dip depth, field localization, angular response, and tolerance analysis provides a more complete assessment of the sensor performance. This is important because practical biosensing requires not only a large resonance shift, but also a sharp, stable, and clearly detectable resonance feature.

**Table 2:** Comparison of various biosensors highlighting sensitivity, Q-factor, and unique features.

Sensor Type / Structure	Material System	Sensitivity	Q-Factor	Special Feature	Reference
SPR Biosensor	Graphene–MoS₂ hybrid	~ 200 °/RIU	Moderate	Nanocomposite enhancement	[24]
SPR Biosensor	MoS₂ monolayer	~ 235 °/RIU	56	2D material integration	[25]
Photonic Crystal Sensor	GST-based PC	175–267 nm/RIU	87.875	Phase-change tunability	^26^
Photonic Crystal Sensor	GaN-based PC	208 nm/RIU	605	Defect-mode resonance	^27^
MoS₂ SPR Sensor	MoS₂ multilayer	261.33 °/RIU	36.16	Biofluid detection	^28^
2D PhCs	2D- microcavity ring	995 nm/RIU	90	malignant cells	^29^
2DPhCs	2D-PhCs	1148 nm/RIU	193	Oral cancer	^30^
1D PC Biosensor	Graphene-assisted 1DPhC	1629.82 nm/RIU	470.25	Oral cancer	Proposed Work

Therefore, the comparison in Table 2 should be understood as an application-specific comparison for optical refractive-index biosensing, particularly for oral-cancer-related detection, rather than a strict universal comparison between different sensor classes.

## Conclusion

In conclusion, a graphene-assisted one-dimensional photonic crystal biosensor for oral cancer detection in the mid-infrared region has been successfully designed and theoretically investigated using the transfer matrix method. The proposed structure supports a highly localized defect-mode resonance inside the photonic band gap, where small refractive-index variations in the oral-tissue cavity produce measurable resonance wavelength shifts. The obtained results demonstrated excellent sensing performance, including high sensitivity, strong linearity, narrow resonance linewidth, and stable optical confinement. The calculated performance parameters confirmed that the proposed design achieves a balanced sensing response characterized by high spectral selectivity and low detection limit rather than relying solely on resonance displacement. Electric-field distribution analysis revealed strong localization of the electromagnetic field inside the defect cavity, explaining the enhanced light–matter interaction responsible for the observed sensing behavior. Moreover, the complementary reflectance and transmittance responses verified the stability and physical consistency of the localized defect mode. Angular analysis, defect-thickness optimization, and fabrication-tolerance evaluation further demonstrated the robustness of the proposed sensor under practical operating conditions. These findings indicate that the proposed graphene-assisted photonic crystal biosensor represents a promising and physically reliable platform for highly sensitive oral cancer detection and may provide useful guidance for future experimental implementation and biomedical photonic sensing applications.

## Data Availability

The datasets used and analyzed in this study are available upon reasonable request from the corresponding author.

## References

[1] Sung, H. et al. Global cancer statistics 2020: GLOBOCAN estimates of incidence and mortality worldwide. *Cancer J. Clin.* **71**, 209–249. 10.3322/caac.21660 (2021). 10.3322/caac.21660 33538338

[2] Mattiuzzi, C. & Lippi, G. Current cancer epidemiology. *J. Epidemiol. Global Health*. **9**, 217–222. 10.2991/jegh.k.191008.001 (2019). 10.2991/jegh.k.191008.001 PMC7310786 31854162

[3] Schiffman, J. D. & Fisher, P. G. Early detection of cancer: Past, present, and future. *ASCO Educational Book.* **35**, 57–65. 10.14694/EdBook_AM.2015.35.57 (2015). 10.14694/EdBook_AM.2015.35.57 25993143

[4] Fass, L. Imaging and cancer: A review. *Mol. Oncol.* **2**, 115–152. 10.1016/j.molonc.2008.04.001 (2008). 19383333 10.1016/j.molonc.2008.04.001 PMC5527766

[5] Albert, J. M. Radiation therapy and oncology advances. *J. Clin. Oncol.* **31**, 138–139. 10.1200/JCO.2012.45.0215 (2013).

[6] Gowda, B. K. et al. Photonic crystal-based biosensor for cancer detection. *Optik*10.1016/j.ijleo.2021.166555 (2021).

[7] Konopsky, V. N. et al. Photonic crystal biosensors. *Biosens. Bioelectron.* **38**, 333–337. 10.1016/j.bios.2012.06.012 (2013).

[8] Fenzl, C. et al. Photonic crystal biosensing. *Anal. Bioanal. Chem.* **406**, 2727–2737. 10.1007/s00216-014-7648-7 (2014).24553662

[9] Threm, D. et al. Photonic crystal biosensors. *J. Biophotonics*. **5**, 601–616. 10.1002/jbio.201100080 (2012). 22678992 10.1002/jbio.201200039

[10] Wu, D. & Qu, F. Surface-enhanced Raman scattering-based biosensors. *Chem. Soc. Rev.* **44**, 2963–2997. 10.1039/C4CS00286C (2015).25739971

[11] Arlett, J. L. et al. Mechanical biosensors. *Nat. Nanotechnol.* **6**, 203–215. 10.1038/nnano.2011.44 (2011). 21441911 10.1038/nnano.2011.44 PMC3839312

[12] Haleem, A. et al. Biosensors applications in medical field. *Sens. Int.* **2**, 100100. 10.1016/j.sintl.2021.100100 (2021).

[13] Joannopoulos, J. D. et al. *Photonic Crystals: Molding the Flow of Light* (Princeton University Press, 2008). 10.1515/9781400828241

[14] Jafari, H. et al. Photonic crystal sensors. *Opt. Mater.* **124**, 111993. 10.1016/j.optmat.2021.111993 (2022).

[15] Panda, A. & Devi, S. Photonic crystal sensor for cancer detection. *Opt. Quant. Electron.*10.1007/s11082-020-02276-8 (2020).

[16] Olyaee, S. & Najafgholinezhad, S. High-performance photonic crystal sensor. *Optik*10.1016/j.ijleo.2013.01.047 (2013).

[17] Seddon, A. B. Mid-infrared photonics. *Nat. Photonics*. **5**, 28–36. 10.1038/nphoton.2010.318 (2011).

[18] Ouardi, M. E. et al. Development of a novel SPR biosensor for early pregnancy detection. *Sens. Imaging* **26**, 56. 10.1007/s11220-025-00582-w (2025).

[19] Ouardi, M. E. et al. Multi-purpose Surface Plasmon Resonance Sensor with Enhanced Sensitivity for Detecting Anemia and Monitoring Glucose Levels. *Plasmonics* **20**, 8253–8266. 10.1007/s11468-025-02813-y (2025).

[20] Sharma, P., Medhekar, S. & Ultra-Compact Highly Sensitive 2D Photonic Crystal L3 Cavity Biosensor. *Sens. Imaging*. **26**, 130. 10.1007/s11220-025-00656-9 (2025).

[21] Aly, A. H. et al. Biophotonic sensor for the detection of creatinine concentration in blood serum based on 1D photonic crystal. *RSC Adv.* **10**, 31765–31772. 10.1039/D0RA05448H (2020). 35518172 10.1039/d0ra05448h PMC9056509

[22] Yeh, P. *Optical Waves in Layered Media* (Wiley, 2005).

[23] Born, M. & Wolf, E. Principles of Optics (7th Edition). Cambridge University Press. (1999).

[24] Vahed, H. et al. Sensitivity enhancement of SPR optical biosensor based on Graphene–MoS₂ structure with nanocomposite layer. *Opt. Mater.* **88**, 161–166. 10.1016/j.optmat.2018.11.034 (2019).

[25] Anil Kumar, A. K., Yadav, A. S., Kushwaha & Srivastava, S. K. A comparative study among WS₂, MoS₂ and graphene based surface plasmon resonance (SPR) sensor. *Sens. Actuators Rep.* **2** (1, 100015, ). 10.1016/j.snr.2020.100015 (2020).

[26] Walaa, M. et al. Chemical-enhanced thyroid cell detection using photonic crystal biosensors with phase-change materials. *RSC Adv.* **16**, 10113–10128. 10.1039/D5RA09237J (2026). 41726223 10.1039/d5ra09237j PMC12917951

[27] N. H. and A. S., Design and analysis of a GaN-based 2D photonic crystal biosensor integrated with machine learning techniques for detection of skin diseases. *Sci. Rep.*, **15**, 41863, (2025). 10.1038/s41598-025-25893-6 10.1038/s41598-025-25893-6 PMC12647168 41290818

[28] Tene, T., Tubon-Usca, G., Gallegos, K. T., Mendoza Salazar, M. J. & Vacacela Gomez, C. MoS₂-based biosensor for SARS-CoV-2 detection: a numerical approach, Frontiers in Nanotechnology, vol. 6, art. 1505751, (2025). 10.3389/fnano.2024.1505751

[29] Harshitha, V. S. & Rohan, R. Design and analysis of optically sensitive biosensor using two-dimensional photonic crystal for oral cancerous cell detection. *J. Opt.* **53**, 4649–4655. 10.1007/s12596-023-01619-7 (2024).

[30] Hossein Ghaforyan. Ultra-sensitive Two-dimensional Photonic Crystal Biosensor for Oral Cancerous Cell Detection. *J. Adv. Biomedical Sci.* **14**, 200–209 (2024). https://api.semanticscholar.org/CorpusID:273882154

[31] Goyal, A. K., Divyanshu, D. & Massoud, Y. Nanophotonic Resonator Assisted Photonic Spin Hall Enhancement for Sensing Application. Scientific Reports, vol. 13, 2023, Article 9292. 10.1038/s41598-023-36417-5 10.1038/s41598-023-36417-5 PMC10247723 37286761

[32] Goyal, A. K., Saini, J. & Massoud, Y. Performance Analysis of Organic Material Assisted Dynamically Tunable Excitation of Optical Tamm State. *Opt. Quant. Electron.* **55**, Article563. 10.1007/s11082-023-04843-4 (2023).

[33] Goyal, A. K., Dutta, H. S. & Pal, S. Design and Analysis of Photonic Crystal Micro-Cavity Based Optical Sensor Platform. AIP Conference Proceedings, vol. 1724, no. 1, Article 020005. (2016). 10.1063/1.4945125

[34] Saini, J., Kumar, A. & Goyal, A. K. Performance Analysis of Chirped Graded Photonic Crystal Resonator for Biosensing Applications. Photonics, vol. 11, no. 12, Article 1173. (2024). 10.3390/photonics11121173

[35] Goyal, A. K., Kumar, A. & Massoud, Y. Performance Analysis of DAST Material-Assisted Photonic-Crystal-Based Electrical Tunable Optical Filter. Crystals, vol. 12, no. 7, Article 992. (2022). 10.3390/cryst12070992

[36] Dutta, H. S., Goyal, A. K. & Pal, S. Analysis of Dispersion Diagram for High Performance Refractive Index Sensor Based on Photonic Crystal Waveguides. Photonics and Nanostructures – Fundamentals and Applications, vol. 23, pp. 41–47. (2017). 10.1016/j.photonics.2016.11.004

[37] Kassa-Baghdouche, L. High-Sensitivity Spectroscopic Gas Sensor Using Optimized H1 Photonic Crystal Microcavities. *J. Opt. Soc. Am. B*. **37** (11), A277–A284. 10.1364/JOSAB.398330 (2020).

[38] Kassa-Baghdouche, L. & Cassan, E. Article 328. *Opt. Quant. Electron.* **51** (10). 10.1007/s11082-019-2040-4 (2019). Sensitivity Analysis of Ring-Shaped Slotted Photonic Crystal Waveguides for Mid-Infrared Refractive Index Sensing.

[39] Kassa-Baghdouche, L. & Cassan, E. Mid-Infrared Refractive Index Sensing Using Optimized Slotted Photonic Crystal Waveguides. Photonics and Nanostructures – Fundamentals and Applications, vol. 28, pp. 32–36. (2018). 10.1016/j.photonics.2017.11.001

[40] Kassa-Baghdouche, L., Boumaza, T. & Bouchemat, M. Optical Properties of One-Dimensional Photonic Crystal Structures for Sensing Applications. *Appl. Phys. B*. **121**, 297–305 (2015).

[41] Kassa-Baghdouche, L., Boumaza, T. & Bouchemat, M. Optical Characteristics of Defect Modes in One-Dimensional Photonic Crystal Structures. Physica Scripta, vol. 90, Article 065504. (2015). 10.1088/0031-8949/90/6/065504

[42] Kassa-Baghdouche, L. & Cassan, E. Performance Enhancement of Slotted Photonic Crystal Waveguides for Mid-Infrared Sensing Applications. Optical and Quantum Electronics, vol. 52, no. 5, Article 260. (2020). 10.1007/s11082-020-02370-0

[43] Goyal, A. K., Kumar, A. & Massoud, Y. Thermal Stability Analysis of Surface Wave Assisted Bio-Photonic Sensor. *Photonics* **9**, 324. 10.3390/photonics9050324 (2022).

